# An Effective Approach for Clustering InhA Molecular Dynamics Trajectory Using Substrate-Binding Cavity Features

**DOI:** 10.1371/journal.pone.0133172

**Published:** 2015-07-28

**Authors:** Renata De Paris, Christian V. Quevedo, Duncan D. A. Ruiz, Osmar Norberto de Souza

**Affiliations:** 1 Grupo de Pesquisa em Inteligência de Negócio—GPIN, Faculdade de Informática, PUCRS, Av. Ipiranga, 6681-Prédio 32, sala 628, Porto Alegre, RS, Brasil; 2 Laboratório de Bioinformática, Modelagem e Simulação de Biossistemas—LABIO, Faculdade de Informática, PUCRS, Av. Ipiranga, 6681- Building 32, Room 602, Porto Alegre, RS, Brasil; Institute for Research in Biomedicine, SPAIN

## Abstract

Protein receptor conformations, obtained from molecular dynamics (MD) simulations, have become a promising treatment of its explicit flexibility in molecular docking experiments applied to drug discovery and development. However, incorporating the entire ensemble of MD conformations in docking experiments to screen large candidate compound libraries is currently an unfeasible task. Clustering algorithms have been widely used as a means to reduce such ensembles to a manageable size. Most studies investigate different algorithms using pairwise Root-Mean Square Deviation (RMSD) values for all, or part of the MD conformations. Nevertheless, the RMSD only may not be the most appropriate gauge to cluster conformations when the target receptor has a plastic active site, since they are influenced by changes that occur on other parts of the structure. Hence, we have applied two partitioning methods (*k*-means and *k*-medoids) and four agglomerative hierarchical methods (Complete linkage, Ward’s, Unweighted Pair Group Method and Weighted Pair Group Method) to analyze and compare the quality of partitions between a data set composed of properties from an enzyme receptor substrate-binding cavity and two data sets created using different RMSD approaches. Ensembles of representative MD conformations were generated by selecting a medoid of each group from all partitions analyzed. We investigated the performance of our new method for evaluating binding conformation of drug candidates to the InhA enzyme, which were performed by cross-docking experiments between a 20 ns MD trajectory and 20 different ligands. Statistical analyses showed that the novel ensemble, which is represented by only 0.48% of the MD conformations, was able to reproduce 75% of all dynamic behaviors within the binding cavity for the docking experiments performed. Moreover, this new approach not only outperforms the other two RMSD-clustering solutions, but it also shows to be a promising strategy to distill biologically relevant information from MD trajectories, especially for docking purposes.

## Introduction

Molecular dynamics (MD) simulation and the insights it offers into protein motion is a powerful technique for understanding the structure and function of biological macromolecules in rational drug discovery [1, 2]. It incorporates flexibility on 3D structures of biological macromolecules by exploring the dynamic behavior of proteins at different timescales. A typical MD simulation may generate above of 10^4^ conformations or snapshots to explore the conformational space of the protein concerned by individual particle motions as a function of time [1]. Although this approach is time-consuming, it provides improved accuracy in the molecular docking process and opens new opportunities for the discovery of novel potential drugs [3]. In this study, the large ensemble of snapshots generated by an MD simulation is called a Fully-Flexible Receptor (FFR) model [4]. Typically, FFR models are used to play docking experiments with accessible ligand libraries, which hold currently at least 10^6^ possible solutions [5–8]. Therefore, the high computational cost involved in using FFR models to perform practical virtual screening in such ligand databases may make it unfeasible. For this reason, new and promising approaches to reduce the dimensionality of FFR models systematically—without losing critical structural information—should be investigated and applied [9].

Clustering has been used in a variety of situations, such as understanding virtual screening results [10], partitioning data sets into structurally homogeneous subsets for modeling [11, 12], and picking representative chemical structures from individual clusters [13–15]. The use of clustering algorithms to group similar conformations is the most appropriate data mining technique to distill the structural information from properties of an MD trajectory [16–18]. In this approach, MD receptor conformations are grouped according to some similarity metric such as Root Mean Square Deviation (RMSD) [13, 19] or Distance Matrix Error *D*_*ab*_ (DME) [17].

Several studies used clustering algorithms to investigate dissimilar behavior on the MD trajectory. For example, Li [11] used RMSD differences and dihedral angles transitions from a small MD trajectory of the HIV-1 integrase catalytic core to create conformational ensembles using the Bayesian clustering method. Li applied the posterior probability and the class cross entropy to identify the optimal number of clusters; however, the quality of clustering was measured by visual inspection. Philips et al. [12] developed a framework to validate the performance and utility of spectral clustering algorithms for studying molecular biopolymer simulations. A more detailed analysis on clustering of MD trajectories using different methods was done by Torda and van Gunsteren [17] and Shao et al. [16]. Torda and van Gunsteren created the distance measure *D*_*ab*_ for clustering an MD trajectory with 2,000 structures applying single linkage and hierarchical divisive algorithms, and they concluded that the divisive algorithm produced satisfactory results when a trajectory configuration is evenly distributed across the conformational space. Shao et al. [16] compared eleven different clustering algorithms to assess the performance and differences between such algorithms based on the pairwise RMSD distance. Shao and co-authors used the clustering metrics to find an adequate number of clusters in ensembles of structures taken from a sieving approach. In this approach, a portion of the data is clustered and the remaining data are added to existing clusters in order to handle very large data sets more efficiently. To assess the advantages of using the sieving approach, Shao et al. [16] performed four clustering experiments and concluded that pairwise RMSD values were able to keep the DB [20] and CH [21] values similar to MD conformations collected at every 10, 20, 50, and 500 ps. This sieved clustering performs well when the pairwise RMSD value is the only metric applied to measure the similarity between structures. However, making use of a sieving approach for identifying similarities from properties of the substrate-binding cavity (such as area, volume, and heavy atoms) may lead to loss or distortion of the relations among the original data and to a biased grouping, if the selection at the first stage is not representative.

Alternative studies generate groups of similar conformations in order to find representative objects that reproduce the original MD trajectory [13, 22]. Nevertheless, the ability to apply a clustering method that is strongly sensitive to a measure of similarity and accurately extracts the most meaningful biological information remains challenging. For instance, Lyman et al. [22] generate sets of reference structures by building histograms of nearest MD structures based on different cutoff distances (RMSD). The authors identify the optimal representative ensemble by comparing the convergence of the MD simulation and the relative populations of the clusters. On the other hand, Landon et al. [13] produce representative MD conformations by mapping the number of cluster representatives at a 1.3 Å cutoff using the CS-Map clustering algorithm on apo and holo N1 X-ray structures. Even though both studies are capable of covering very different portions of the conformational space of different MD trajectories, the pairwise RMSD distances remain the only measure of similarity applied. Further, they conduct the experiments with a reduced MD trajectory, which is generated by choosing the smallest observed distance between any pairs of structure based on cutoff values.

**Fig 1:**
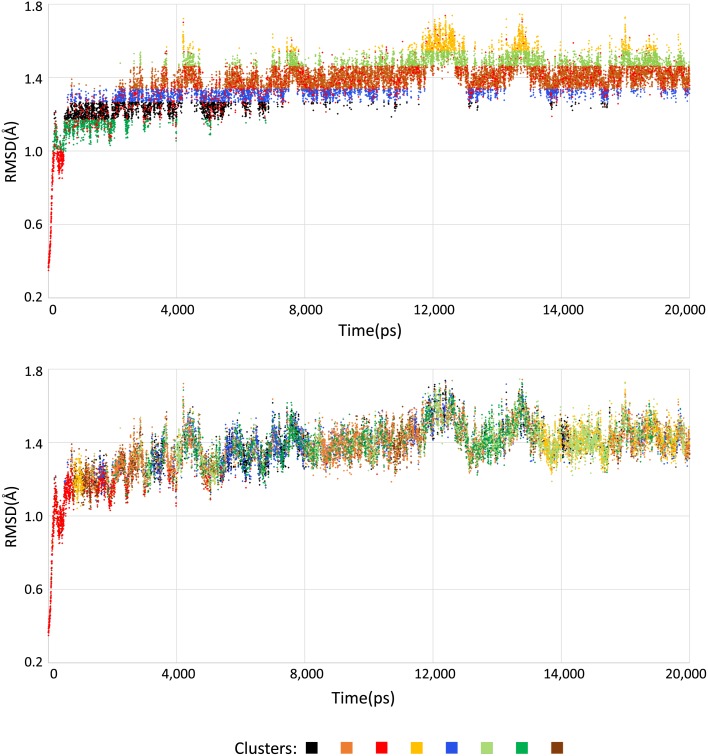
Comparisons of clustering results relative to two different measures of similarity for the InhA enzyme MD trajectory. In each case, the *k*-means clustering algorithm with the number of clusters of eight was used. The difference of the cluster dispersion between the traditional method and our methodology is evidenced by using the pairwise RMSD distance and the properties from the substrate-binding cavity in the scatter plots (a) and (b), respectively.

In contrast to previous works, we concentrate our efforts on identifying small and localized changes that are expected to have a major influence on the interactions between flexible receptors and different ligands. The method we introduced may group similar behavior in the substrate-binding cavity of every MD conformation, which is impossible when using traditional clustering methods, as shown in Fig 1. This figure highlights the differences in the cluster distribution between a traditional RMSD-based clustering (Fig 1a) and the strategy that we are proposing (Fig 1b). The latter depicts alternatives groups of binding modes that the MD simulation holds at different timescales when several attributes from the binding cavity (such as area, volume, RMSD and heavy atoms) are used as input for the *k*-means clustering algorithm. However, because of the pairwise RMSD distance applied as attribute for clustering the partitioning from Fig 1a, the groups of conformations appears strongly influenced by structural changes that occurs along the MD trajectory.

This study presents two main contributions. First, we provide a detailed comparison of six clustering algorithms applied to three different data sets from an MD trajectory. Subsequently, we identify ensembles of reduced and representative MD conformations from the best clustering solutions based on measures of dispersions of estimated Free Energy of Binding (FEB) values by docking experiments performed on AutoDock4 [23]. Towards this end, we compared resulting partitions of every data set, which contain features extracted from a 20 ns MD trajectory of the InhA-NADH complex. The applied algorithms are partitioning methods (*k*-means [24] and *k*-medoids [25]) and agglomerative hierarchical methods (Complete linkage, Ward’s and Group average agglomerative methods). A performance comparison was made among two data sets formed by different pairwise RMSD-based approaches and a data set built with properties from the substrate-binding cavity, which is our novel approach for clustering MD trajectories. The analysis of the generated partitions were conducted by taking into account the representative object of each partition, i.e. the medoids. To select the best partitions, we assess quartile values from medoids of each partition based on predicted FEB values, which were obtained by performing cross-docking experiments involving protein-ligand complexes with 20 different ligands and the FFR model under study. Quartile values are computed in a detailed progression of the partitioning, thereby allowing characterization of clustering performance across a range of number of clusters. Ensembles of representative MD snapshots were selected from the best partitioning performance to evaluate the quality of the proposed ensemble. To illustrate this, we evaluated if such a small and representative ensemble, which holds less than 0.4% of all conformations, is able to cover a high level of dissimilar binding modes that the full set of MD conformations can assume when it is submitted to docking experiments. Consequently, we expect to significantly reduce the redundancy in the full set of conformations, and thus make computationally tractable the practice of performing virtual screening experiments on MD trajectories, without losing the most biologically relevant information.

This paper is structured as follows. Section 2 describes details from the MD simulation, the structural features from the substrate cavity of every MD conformation, clustering methods and statistical metrics used to evaluate the quality of the produced clustering. Section 3 reports the analyses and experiments performed to group MD structures from three different data sets and six clustering algorithms as well as presents the reference structures selected to span the whole MD trajectory. Finally, Section 4 describes the conclusions and future work directions.

## Materials and Methods

### Molecular Dynamics (MD) simulation

The MD trajectory used in this study was generated from the 2.2 Å crystal structure of the enzyme Enoyl-Reductase or InhA-NADH complex from *Mycobacterium tuberculosis* (PDB ID: 1ENY) [26] with 41 crystallographic water molecules as described in Gargano et al. [27]. In this simulation, the Amber 9 program suite [28] and the AMBER ff99SB force field [29] were used to extract data at every 1 ps interval over the 20 ns simulation, yielding a set of 20,000 instantaneous receptor structures, being also referenced as a FFR model of InhA. For the NADH molecule the atomic charges were assigned by *ab initio* calculations in the HF 6-31G* level [30] and fitted with the RESP procedure [31], which are fully compatible with the AMBER force field [29]. The structures belonging to the FFR model were superimposed onto the initial structure using a least-square fit and the protein was solvated with 10,491 TIP3P water molecules in a rectangular box of 77.7 Å x 73.3 Å x 77.3 Å. All hydrogen atoms, ions and water molecules were initially submitted to 100 steps of energy minimization with steepest descent in order to remove closely contacts of van der Waals forces. The pressure of the simulation was kept at 1 atm and, in order to avoid disturbance to the system, the temperature was gradually increased from 10 K up to 298 K in six steps (10 K to 50 K, 50 K to 100 K, and so forth). For these steps of temperatures, the velocities were reassigned according to the Maxwell-Boltzmann distribution and equilibrated for 200 ps.

We emphasize the changes that the macromolecular structure suffers during an MD simulation. The RMSD of protein backbone atoms from the initial structure is often used as an indicator of structural changes, as shown in Fig 1. The plot displays the structural variation of the InhA’s full trajectory and delineates the equilibration phase at 500 ps. During the equilibration stage, the results present significant variations because the initial structure is not within the equilibrium phase of the simulation conditions. After this initial stage, MD properties can be efficiently studied by keeping the system in a steady non-equilibrium state [32]. For this reason, the first 500 conformations were withdrawn from the MD trajectory before starting the clustering analyses. The RMSD variation of the production phase is stabilized between 1.0 Å and 1.8 Å, reaching a plateau around 1.4 ± 0.1 Å.

### Extracting structural features from the MD trajectory

For clustering the MD conformations, we generated the following data sets:

1. Protein RMSD. It has the pairwise RMSD distance between the first and every MD’s structure, considering all structure residues as applied by [16, 22, 33] (S1 Dataset).
2. Cavity RMSD. This data set contains the RMSD distance between the first and every MD’s structure, considering the residues that enclose the substrate-binding cavity of the InhA enzyme in complex with NADH and the C_16_ substrate analog (PDB ID: 1BVR) enzyme. Application examples of this measure of similarity are in [13, 34] (S2 Dataset).
3. Cavity Attributes. It was built by using a set of features extracted from the substrate-binding cavity of the MD trajectory. This is the proposed data set and a more detailed explanation is given in this section (S3 Dataset).

The first two data sets were generated from typical measures of similarity for clustering MD simulations. Our purpose in using these data sets is to compare the quality of partitions between them and the Cavity Attributes data set.

For generating the Cavity Attributes data set, we extract structural properties from the substrate-binding cavity of each conformation generated by an MD simulation. From those features, we seek to partition dissimilar behaviors found within the binding site along an MD simulation followed by generating an ensemble of representative structures that allows the covering of localized protein movements to improve the fitting of ligands during the docking process. The structural features extracted from the substrate cavity of each FFR model’s conformation and used as input to the clustering algorithms are:

1. the volume of the substrate cavity (in Å³);
2. the number of heavy atoms present in the substrate-binding cavity of the 1BVR structure [35]; and;
3. the pairwise RMSD distance relative from the first to the current snapshot (in Å).

Pairwise RMSD distances were evaluated by using the differences among the backbone C*α* atoms from the first structure against the conformation being compared, using the following equation:

$$RMSD_{r_{t},r_{ref}}=\sqrt{\frac{1}{n}\overset{n}{\underset{i=1}{\sum }}|r_{t,i}-r_{ref.i}{|}^{2}}$$ (1)

where r_t,i_ and r_ref,i_ are the positions of equivalent atoms in the conformation at time t(r_t_) along the MD simulations and the reference structure (r_ref_), respectively. The RMSD was calculated using the *ptraj* module from AmberTools14 [36].

**Fig 2:**
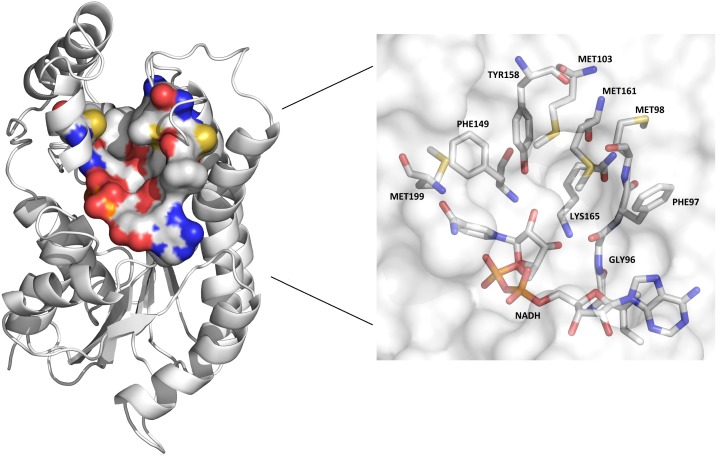
Substrate-binding cavity of the InhA enzyme (PDB ID: 1BVR) identified by the CASTp software tool. On the left, the substrate-binding cavity of the 1BVR structure represented by molecular surface and colored by atom types (carbon and hydrogen: light grey; nitrogen: blue; oxygen: red; sulphur: yellow). The projection displays all residues from the binding pocket in stick representation.

The remaining features were taken from CASTp’s results [37]. CASTp is an online software tool that allows us to obtain information from all cavities in a structural manner through a free access to the source code of the results page. It relies on the alpha-shape method [38] to enclose the substrate cavity on proteins. This method uses the solvent-accessible surface area model [39] and the molecular surface model [40] with a probe sphere of radius 1.4 Å. To identify the substrate cavity on an ensemble of conformations generated by MD simulation, we developed a heuristic function based on the number of heavy atoms present in the substrate-binding cavity of the 1BVR structure [35]. The substrate analog, which is inside the 1BVR crystallographic structure, allowed us to identify the substrate cavity and the largest number of atoms, considering the residues that encloses it. Thus, we calculated the volume and the number of heavy atoms of the substrate cavity for each snapshot based on the substrate analog, according to the cavities present in the 1BVR and selected by CASTp. Fig 2 shows the substrate cavity of the 1BVR structure identified by the CASTp software along with the residues that enclose the substrate-binding cavity, which are GLY96, PHE97, MET98, MET103, PHE149, TYR158, MET161, LYS165, MET199, NADH (coenzyme).

The volume from the substrate-binding cavity was chosen as one of attributes from Cavity Attributes data set since it varies considerably along the MD simulation (S1 Fig). This is evidenced by analyzing the substrate-binding cavity volumes generated by CASTp, which ranged from 45.4 Å^3^ to 2,852.9 Å^3^ for the entire 20 ns MD simulation trajectory. We also note that the volumes of the substrate-binding cavity from the MD trajectory comprise proportionally those found in the boundaries of the InhA crystal structure. For instance, cavity volumes from 2B37 and 4OXN structures are 445.1 Å^3^ and 2,032.8 Å^3^, respectively, pointing out to significantly different volume values in the MD trajectory.

Although the volume allows us to identify the biggest accessible surface of the substrate-binding cavity, we also considered the geometric properties of the cavity to identify whether or not the cavity dimension allows the fitting of a ligand within this 3D space. For that, we developed a function based on a weight system. It gives weight 1 for the atoms whose residues determine the substrate analog in the crystal structure, and 3 for atoms whose residues surround the NADH nicotinamide ring. Remember that the substrate cavity is placed on the NADH coenzyme, which shapes the base of the target cavity and, for this reason, has more weight. The algorithm output is a set of cavities and their respective scores (sum of weights) for every conformation. Hence, we consider cavities with potential level of binding those that show high scores of weights.

The number of heavy atoms summarized by the residues from the binding cavity are illustrated in Table 1. In this table, it is clear that the RMSD values are fully sensitive to conformational changes. However, the direction of internal motions is only detected by the volume and heavy atoms, which in turn recognizes small and localized changes that take place in the solvent-accessible surface of flexible systems. For instance, the first two and last two structures contain equal RMSD values; however there is a considerable difference between the volume value and the number of heavy atoms for each residues. The values from Table 1 are in different ranges since they correspond to the original information of each conformation. For clustering purposes, we created a CSV file with the data normalized within the interval [0,1]. It is noteworthy that this methodology is not specific for the InhA’s protein; it may be used for any structure that contains its binding sites known in advance.

**Table 1:** Fragment of the Cavity Attributes data set used for clustering the MD trajectory. The first line labels the substrate-binding cavity features. The number below each residue indicates the maximum number of heavy atoms it can hold.

RMSD	Volume	GLY96	PHE97	MET98	MET103	PHE149	TYR158	MET161	LYS165	MET199	NADH
(Å)	(Å^3^)	(4 atoms)	(11 atoms)	(8 atoms)	(8 atoms)	(11 atoms)	(12 atoms)	(8 atoms)	(9 atoms)	(8 atoms)	(9 atoms)
0.37	607.00	2	4	3	2	6	4	3	2	6	6
0.37	795.10	2	4	3	2	5	3	2	0	6	6
…	…	…	…	…	…	…	…	…	…	…	…
1.39	1551.40	3	8	5	4	2	4	4	3	1	5
1.38	929.80	2	6	4	3	2	5	3	3	1	5
1.38	516.90	3	4	3	2	2	4	3	2	2	6

### Clustering Algorithms

The large ensemble of MD conformations was clustered using algorithms implemented in the R Programming Language [41]. *k*-means [24], *k*-medoids [25], agglomerative hierarchical [25] methods and their variations were used to find representative clusters of the FFR model. *k*-means and *k*-medoids belong to the set of partitioning clustering methods, which divide a set of data objects into non-overlapping subsets with spherical shape such that each data object is in exactly one subset [42, 43].

*k*-means is a well-known clustering algorithm that locally optimizes the average squared distance of points from their nearest cluster center (centroid). It randomly chooses *k* centroids, and refines them throughout several iterations, where the distance of every point to the *k* centroids are computed to determine the cluster memberships [24]. To generate groups more compact and separate as possible, the *k*-means algorithm applies the sum of squared errors (E_Means_) between all objects p of a given cluster C_h_ and its centroid c_h_ for all clusters k according to the following equation:

$$E_{Means}=\overset{k}{\underset{h=1}{\sum }}\overset{}{\underset{p\in C_{h}}{\sum }}dist{\left(p,c_{h}\right)}^{2}$$ (2)

In contrast to *k*-means, whose centroid almost never correspond to an object, *k*-medoids uses the PAM (Partitioning Around Medoids) algorithm [25] for clustering data sets based on central objects. This algorithm chooses a set of representative objects or medoids to determine whether a non representative object is a good replacement for a current medoid [43]. While the *k*-means technique uses the sum of the squared error function to measure the within-cluster variation, the *k*-medoids algorithms apply an absolute error criterion. In this method, the objects (*n*) are grouped into *k* clusters by minimizing the sum of the dissimilarities between each object and its corresponding representative. Then, the sum of the absolute error for all objects *p* in the data set is defined as:

$$E_{Medoids}=\overset{k}{\underset{h=1}{\sum }}\underset{p\in C_{h}}{\sum }dist(p,o_{h})$$ (3)

Where o_h_ is the representative object of C_h_. PAM is the algorithm used to compute medoids for small data sets. To deal with large data, PAM has an extension called CLARA (Clustering LARge Applications) [25]. CLARA optimizes the k-medoids performance by generating samples from the entire data set and computing the medoids from them using PAM algorithm. Even though CLARA is used to reduce the time taken to generate partitions from k-medoids, it is still a time-consuming task since its complexity is O(ks^2^+(k(n-k))), where *s* is the size of the sample, k is the number of clusters and n the number of objects. In this study, CLARA is the algorithm applied to generate the *k*-medoid partitions due to the dimension of our data sets.

Unlike partitioning clustering, hierarchical clustering methods aim to group data into levels such as in a hierarchy or “tree” of clusters [43]. It has two basic approaches, known as agglomerative and divisive. The agglomerative hierarchical clustering, which uses the bottom-up strategy, starts with each object as an individual cluster and interactively merges the closest pair of clusters until all the objects are in a single cluster or the maximum number of clusters is reached. The divisive hierarchical clustering, which uses the top-down strategy, starts with all objects in the same cluster and splits a cluster into smaller clusters in each iteration until each object becomes a singleton cluster or a termination condition holds [42, 43]. In this study, we use only the agglomerative algorithms since the divisive method does not handle efficiently large data sets due to its computational costs. The limiting factor of the divisive method is that there are 2^n−1^−1 possible ways to partition a set of n objects into two subsets.

To measure the proximity between two points in two different clusters, the agglomerative algorithms widely use the methods known as single linkage, complete linkage, median, centroid, group average and Ward’s. In our study, all these methods were applied for clustering the data sets using the AGNES (AGglomerative NESting) method [25]. However, there are certain drawbacks associated with the use of some agglomerative algorithms. Two of these are the high number of singleton clusters and sensibility to outliers. For instance, we found in some partitions more than 50% of all MD conformations within a single large cluster. Hence, we identified that the best agglomerative methods to adopt for this investigation are Complete Linkage, Unweighted Pair Group Method using Arithmetic averages (UPGMA), Weighted Pair Group Method using Arithmetic averages (WPGMA) and Ward’s.

The complete linkage version of hierarchical clustering tends to minimize the increase in diameter of the clusters at each iteration by determining the proximity of two clusters (C_i_, C_j_) as the maximum distance based on the following equation:

$$Complete(C_{i},C_{j})=\underset{x\in C_{i},y\in C_{j}}{\text{max}}dist\left\{\left|x-y\right|\right\}$$ (4)

where |x-y| is the distance between two objects or points *x* and *y*.

In UPGMA and WPGMA, which are group average agglomerative methods, the distance between two clusters is defined as the average pairwise proximity among all pairs of points or objects in different clusters. The difference between these methods is the weight given to the points in different clusters to measure the pairwise proximity. While UPGMA takes into account the number of points in each cluster making a linkage between groups, WPGMA treats all clusters equally making a linkage within groups. The equations defined to measure the distance between the clusters for UPGMA and WPGMA methods are:

$$UPGMA(C_{i},C_{j})=\frac{1}{n_{i}n_{j}}\underset{x\in C_{i}}{\sum }\underset{y\in C_{j}}{\sum }dist|x-y|$$ (5)

$$WPGMA(C_{i},C_{j})=\frac{1}{2}\underset{x\in C_{i}}{\sum }\underset{y\in C_{j}}{\sum }dist|x-y|$$ (6)

where n_i_ and n_j_ are the number of objects from cluster C_i_ and C_j_, respectively, and |x-y| is the distance between two objects or points *x* and *y*.

The Ward’s method, also called the minimum variance method, aims to merge pairs of clusters with minimum variance. It evaluates how much the sum of squares will increase when two clusters are merged. The “merging cost” of combining the clusters is defined as:

$$Ward=\overset{k}{\underset{h=1}{\sum }}\underset{x_{i}\in C_{h}}{\sum }\overset{p}{\underset{j=1}{\sum }}dist{\left(x_{ij}-{\bar{x}}_{{}_{hj}}\right)}^{2}$$ (7)

$${\bar{x}}_{hj}=\frac{1}{n_{h}}\sum_{x_{i}\in C_{h}}x_{ij}$$ (8)

where x_ij_ denotes the value for the i^th^ individual in the *j*-cluster, k is the total number of clusters at each stage, and n_j_ is the number of individuals in the j^th^ cluster. As hierarchical agglomerative clustering method starts defining each data point as its own cluster, the sum of squares begins at zero and grows as the algorithm merge clusters. At each state, this growth is regulated by the Ward’s method, which in turn seeks to merge clusters with the smallest sum of squares.

There are several comparative studies reported in the literature that evaluate the clustering methods described in this section and apply them to different data set types [25, 44, 45]. According to Jain and Dubes [45], there is no perfect clustering algorithm that assures the best solutions for all data sets. The exploratory analysis and understanding on data sets are decisions as important as to the selection of the strategy (such as number of clusters, prototype and clustering method) to be adopted [45].

### Evaluating the data partition

**Fig 3:**
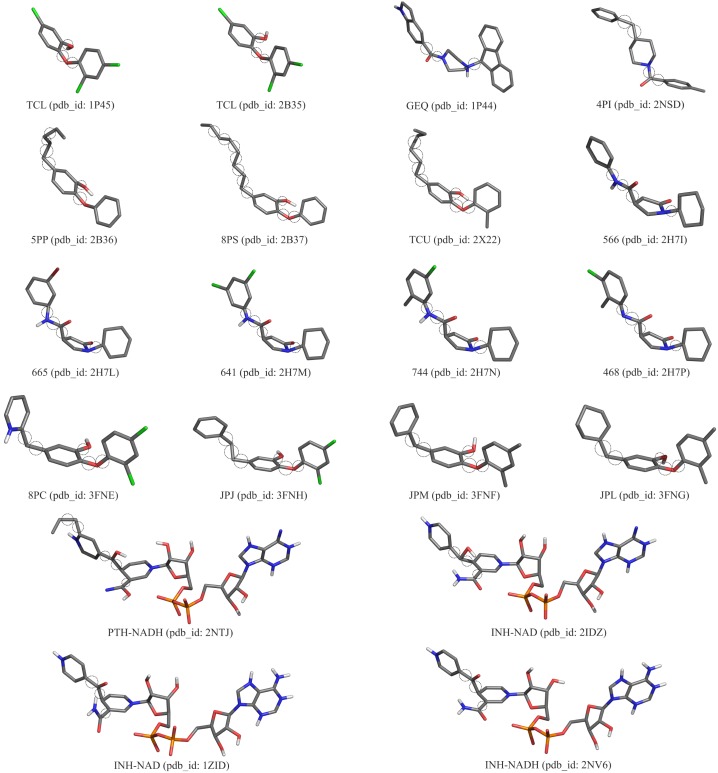
3D structure of the 20 ligands used in docking experiments. Each ligand is colored by atom type (carbon and hydrogen: light grey; nitrogen: blue; oxygen: red; chloro: green; phosphorus: orange; sulphur: yellow) and displays its name and PDB identification (PDB ID). The dashed circle represents the rotatable bounds selected by AutoDockTools 1.5.6. Ligand Abbreviations: **TCL**: triclosan; **665:**(3S) N-(3-bromophenyl)-1-cyclohexyl-5-oxopyrrolidine- 3-carboxamide; **566:**(3S)-1-cyclohexyl-5-oxo-N-phenyl pyrrolidine-3-carboxamide; **8PC:** 2-(2,4-dichloro-phenoxy)-5-(pyridin-2-ylmethyl)phenol; **JPJ:** 2-(2,4-dichlorophenoxy)-5-(2-phenylethyl)phenol; **JPL:** 5-(cyclohexa-1,5-dien-1-ylmethyl)-2-(2,4-dichlorophenoxy) phenol; **JPM:** 5-benzyl-2-(2,4-dichloropheno-xy)phenol; **468:**(3S)-N-(3-chloro-2-methylphenyl)-1-cyclohe xyl-5-oxopyrrolidine-3-carboxamide; **641:**(3S)-1-cyclohexyl-N-(3,5-dichlorophenyl)-5-oxopyrrolidine-3-carboxamide; **744:** (3S)-N-(5-chloro-2-me-thylphenyl)-1-cyclohexyl-5-oxopyrrolidine-3-carboxamide; **INH-NAD:** Isoniazid + NADH coenzyme; **5PP:** 5-pentyl-2-phenoxyphenol; **8PS:** 5-octyl-2-phenoxyphenol; **TCU:** 5-hexyl-2-(2-methylphenoxy)- phenol; **PTH-NAD:** Prothionamide + NADH coenzyme; **THT:** trans-2-hexadecenoyl-(n-acetyl-cys-teamine)- thioester **4PI:** N-4-methylbenzoyl-4-benzylpiperidine; **GEQ:** 5-{[4-9H-fluoren-9-YL)pipera- zin-1-YL]carbonyl}-1H-indole.

The proposed data set, which uses properties from the substrate-binding cavity of the MD trajectory, and the other two RMSD-based data sets were submitted to six different clustering methods and their results were compared. For each data set different seed values for *k*-means and *k*-medoids were configured and Complete linkage, UPGMA, WPGMA and Ward’s methods from agglomerative hierarchical clustering methods were applied to generate the partitions of MD conformations. The quality of partitions were assessed by computing the first, second and third quartiles values of the medoids for the number of clusters ranging from 10 to 200. Quartiles are robust statistic measures capable of indicating central tendency and dispersion of the data points inside a data set, being also resistant to outliers. As we seek to identify partitions that contain MD conformations with high affinity in their binding mode, we investigate the performance of our resulting partitions for evaluating binding conformations of different drug candidates to the enzyme under study. In this regard, 20 compounds experimentally tested (Fig 3) were used in the cross-docking experiments against the FFR model to predict their lowest energy bound conformation (i.e. pose). This set of ligands was extracted from 20 InhA crystal structures available on Protein Data Bank (PDB) [46]. We decided to use ligands from the available InhA crystallographic structures since the overall topology RMSD of their complexes are significantly different. According to Pauli et al. [47] the binding cavity volumes of the 36 InhA Crystal structures available on PDB ranges from 1,597.3 Å^3^ to 3,046.7 Å^3^ for the whole cavity (NADH + substrate-binding cavity). This range represents the different sizes and structures of the ligands that are bound in such structures, being a good sample of structures to dock in our MD trajectory, which has its binding cavity volume ranges from 419.4 Å^3^ to 2,032.8 Å^3^ (InhA-NAD complex).

The docking experiments were performed on FReMI [48], which is a powerful middleware developed to execute molecular docking simulations of FFR models in multiprocessing machines using the AutoDock4.2.5.1 software [23]. We set up AutoDock4.2 to run the Lamarckian Genetic algorithm on three Intel Core i7-2600 3.4 GHz computers with 12 GB RAM, using as operating system the Linux Ubuntu version 13.04. The number of docking runs was set up to 25 since we are working with flexible structures. The box dimensions for grid parameters were tailored according to the structure of each ligand. We defined the atom types of AutoDock, add the Gasteiger charges and merge the non-polar atoms for each snapshot of the FFR model. All these configurations were determined before running the docking experiments and the input AutoDock files were generated during the experiments by FReMI [48] based on AutoDockTools 1.5.6 parameters. The energy evaluation and the number of generations were configured as 300,000 and 27,000, respectively. All ligands were treated as flexible in AutoDock and their rotatable bounds are highlighted in Fig 3. In order to preserve the environmental conditions available to the substrate and ligands, the NADH coenzyme was considered as part of the protein receptor. Conversely, we removed the NADH coenzyme from all snapshots of the FFR model when we performed the experiments with adducts (INH-NAD and PTH-NAD), since they already have the coenzyme as part of their structures.

To obtain ensembles of representative MD conformations from the clustering algorithms used we assessed the dispersion of the resulting partitions based on the predicted FEB values. The dispersion corresponds to the first, second and third quartile values that were calculated to compare the level of convergence between the resulting partitions and the MD’s full trajectory. The first step in this process was to extract the medoids from all partitioning and obtained an ensemble of medoids for every partitioning. This provided us diverse reduced and representative ensembles of MD conformations. The second step was to calculate the quartile values from the ensembles of reduced MD conformations (or medoids) as follow:

$$Quartiles=\overset{N}{\underset{i=1}{\sum }}\overset{Q}{\underset{j=1}{\sum }}x_{ij}$$ (9)

where N is the amount of ligands and Q identifies the first, second and third quartiles, which were measured based on the predicted FEB values obtained from cross-docking experiments. With the quartile values at hand, we then evaluated the Sum of the Quartile Differences (SQD) in order to identify ensembles of medoids having similar dispersion to the MD’s full trajectory. Thus, the SQD values were taken based on the following equation:

$$SQD=\left|\left({\bar{xq}}_{1}-{\bar{yq}}_{1})\left|+\right|({\bar{xq}}_{2}-{\bar{yq}}_{2})\left|+\right|({\bar{xq}}_{3}-{\bar{yq}}_{3}\right)\right|$$ (10)

$${\bar{xq}}_{j}=\frac{1}{n_{i}}\underset{x_{i}\in Q_{j}}{\sum }x_{ji}$$ (11)

$${\bar{yq}}_{j}=\frac{1}{n_{i}}\underset{x_{i}\in Q_{j}}{\sum }y_{ji}$$ (12)

where x_ji_ and y_ji_ denotes the value for the j ligand in the i-quartile and Eqs (11) and (12) detail the means of quartiles calculated for medoids and the MD’s full trajectory, respectively. Low SQD values indicates ensemble of representative MD conformations with high similarity of dispersion to the MD’s full trajectory.

In this study, we analyzed partitions from three different data sets and six different clustering methods. Even though we are using FEB predicted values to select and validate the optimal partitions, we believe that our methodology can provide an effective strategy for improving clustering MD trajectory approaches. Further, we expect that the best set of medoids can also be able to reproduce a fairly good level of distinct binding models for different ligands—especially when the Cavity Attribute data set is used as input for the clustering algorithm.

## Results

One of the major challenges in performing docking experiments of FFR models is the computational demand to screen large databases of small compounds and extract potential binders. According to Amaro et al. [9], performing virtual screening experiments in the full set of structures is computationally intractable and likely unnecessary. Zhong et al. [49] and Cheng et al. [50] show that using a minimal representative ensemble of MD conformations is a promising way to reduce the number of docking experiments and predict high ligand-binding affinity in the ensemble of receptor conformations. For instance, Zhong et al. [49] compared the docking results between the crystal structure and the representative ensemble of five conformations from an MD trajectory with 1,000 snapshots, and concluded that around 90% of active compounds discovered were chosen based on MD-generated representative clusters. Another similar approach is applied by Cheng et al. [50], which distill the three dominant configurations from the MD simulations of avian influenza N1 neuraminidase in the apo form and in complex with the inhibitor oseltamivir. They performed virtual screening with the representative structures and the docking results (FEB values) were validated using the relaxed complex scheme.

The hypothesis we try to confirm in this paper is that the methodology used for clustering the MD trajectory can distill its most meaningful substrate-cavity binding information more effectively. Specifically, we seek to reduce the computational time of using a very large MD trajectory, i.e., more than thousands of conformations, to perform virtual screening of thousands or millions of ligands. One way to address this issue is to create minimal representative ensembles by selecting an MD conformation of each cluster (i.e. a medoid) from a suitable partition. With this in mind, we analyze if the use of clustering algorithms can help us to find relationships between the interactions of FFR models and ligands. Thus, we concentrate efforts on using clustering methods and check their results in order to validate our working hypothesis. Our main contribution is on investigating clustering algorithms to find similarities among snapshots from an MD simulation in order to reduce the FFR model dimension to a manageable size, without losing its biologically relevant information. For this purpose, we apply six different clustering methods to group similar snapshots of the FFR model. Then, we analyze their results by evaluating the data distribution of each clustering, taking into account the best FEB results predicted, by performing cross-docking experiments between the whole MD trajectory and the 20 compounds tested experimentally.

### Cross-Docking Experiments

Unlike other studies, which generate ensembles of representative MD conformations by selecting the most variable structures based on RMSD distance [13], we take into account extra features from the substrate-binding cavity to create partitions with high affinity in their clusters. In this work, the level of dispersion among the clusters is evaluated through the SQD (Eq 10) from all partitions generated, using the estimated FEB values. Towards this end, we performed large cross-docking experiments taking inhibitors from 20 crystallographic structures of InhA (Fig 3) and docking them to the FFR model. The lower FEB values equivalent for these docking experiments were taken to compute the partition dispersions from the resulting clustering. Using this method, we seek partitions capable of detecting those binding modes that can be considered for performing virtual screening of libraries of potential ligands.

Table 2 describes the redocking results and summarizes the cross-docking experiments for the ligands used. Redocking experiments were performed to be used as benchmark to assess the quality enhancements by using the FFR model of InhA (20,000 snapshots). Overall, cross-docking experiments present FEB values close and, for some ligands, higher than redocking experiments, as in the case of TCL300, 566, 5PP, 8PS, PTH-NAD, THT and INH-NAD. In addition to FEB, we also considered the RMSD values. This index verifies whether docking parameters specified in the input file are capable of reproducing the interaction and the structure of a known complex [23]. The best results are achieved when the predicted position by the docking algorithm with the lowest energy has the RMSD value less or equal to 2.0 Å from the crystallographic position of the ligand [51]. Table 2 highlights the RMSD values for 665, 468, 641, 744, 8PS, and GEQ since these ligands present energetically favorable interactions with the MD trajectory, but their final binding-mode are significantly different from those obtained by the crystallographic structures.

**Table 2:** Summary of docking experiments performed to analyze the clustering results. The sixth column highlights the cross-docking experiments that showed RMSD values above 2.0 Å for the best FEB values (kcal/mol). For comparison, the third and fourth column indicates docking results (FEB and its corresponding RMSD) obtained by reproducing the original pose of the ligand in its crystal structure.

PDB ID	Ligand	Redocking		Cross-docking		Best FEB	Best FEB
		FEB	RMSD	Best FEB	RMSD	Average	Std. deviation
1P45	TCL400	-8.7	0.7	-7.8	0.4	-5.5	0.5
2B35	TCL300	-6.8	0.5	-8.5	1.5	-6.2	0.6
2H7L	665	-10.5	0.9	-9.7	**2.3**	-7.0	0.6
2H7I	566	-8.7	1.2	-8.9	1.7	-6.5	0.5
3FNE	8PC	-9.9	1.1	-9.6	1.8	-6.9	0.5
3FNH	JPJ	-10.2	1.3	-9.9	1.8	-7.0	0.6
3FNG	JPL	-10.4	0.5	-9.9	1.6	-7.2	0.7
3FNF	JPM	-10.0	0.4	-9.1	1.9	-6.3	0.8
2H7P	468	-9.5	0.7	-9.0	**2.5**	-6.6	0.6
2H7M	641	-9.8	0.8	-9.1	**2.4**	-6.8	0.6
2H7N	744	-10.1	0.9	-9.0	**2.6**	-6.5	0.6
1ZID	INH-NAD	-10.9	2.0	-10.9	0.8	-7.0	0.9
2B36	5PP	-7.9	1.0	-8.9	1.4	-6.2	0.6
2B37	8PS	-7.3	0.9	-8.8	**2.9**	-6.1	0.7
2X22	TCU	-9.9	0.5	-8.3	1.4	-5.9	0.6
2NTJ	PTH-NAD	-10.3	1.9	-11.8	0.8	-6.1	0.9
1BVR	THT	-5.8	1.7	-10.7	1.9	-8.1	0.7
2NSD	4PI	-11.0	0.8	-10.2	2.0	-6.8	0.7
1P44	GEQ	-11.6	0.5	-10.7	**2.8**	-6.4	1.6
2IDZ	INH-NAD	-8.3	2.8	-10.2	0.7	-6.4	0.9

It is worth notice that the FEB and RMSD values from Table 2 show that ligands resulting from adducts of NADH fit better in the FFR model than their crystallographic structures. For instance, RMSD values from the lowest energy conformation for INH-NAD and PTH-NAD ligands are around 0.8 Å in cross-docking experiments and over 1.9 Å in redocking experiments. This well fit is justified by the fact that the FFR model was generated from an MD simulation of the InhA-NADH enzyme complex, which in turn provides suitable clefts in the substrate-binding cavity due to its flexibility. Remaining ligands were unable to overcome RMSD values undertaken by crystallographic structures but they present very similar FEB values. It means that, the FFR model of 1ENY was able to produce a favorable interaction with the ligands even when the RMSD is higher than the crystallographic conformation.

In this study, we omitted details on the level of accuracy of docking experiments since our focus is to employ FEB values predicted from cross-docking experiments and to analyze them to identify optimal partitioning solutions from the clustering methods used. Redocking experiments were performed to take the input docking parameters for cross-docking experiments. The statistical analysis, i.e. average and standard deviation, represents FEB variations predicted by AutoDock4 along the production phase of the MD trajectory against each of the 20 ligands. From Table 2, we can concluded that, except for GEQ ligand, the variation of the FEB values in the cross-docking experiments was less than 0.9 kcal/mol in 68% of the MD conformations, concentrating a large quantity of conformations closely to the average FEB values.

### Clustering analyses on data sets from the MD trajectory

This section reports and compares the results obtained for clustering three different data sets from structural information of the FFR model. We applied the six clustering algorithms described in the Materials and Methods section. In this regard, we first executed the clustering algorithms for Cavity Attributes, Cavity RMSD, and Protein RMSD data sets varying the number of clusters from 10 to 200, and afterward we extracted the medoids from every generated partitioning. Solutions were evaluated based on statistical assessments in the predicted FEB values. We decided to start the clustering analyses from 10 since low *k* values shows poor level of scatter and, consequently are unable to reflect all possible movements of a 20 ns MD trajectory. In opposition, high numbers of clusters tend to represent better dispersion but we limit the cluster ranges up to 1% of all MD conformations since our findings show the best partitioning solution used cluster count less than 100. Our first set of experiments was performed with a number of clusters range from 2 to 1,000. However, we decided to decrease this range for two reasons: (i) the time consuming taken for performing practical virtual screening of large database of ligands in an ensemble with 1,000 representative MD conformations; and (ii) the high level of accuracy achieved by using a representative ensemble with 200 MD conformations.

**Fig 4:**
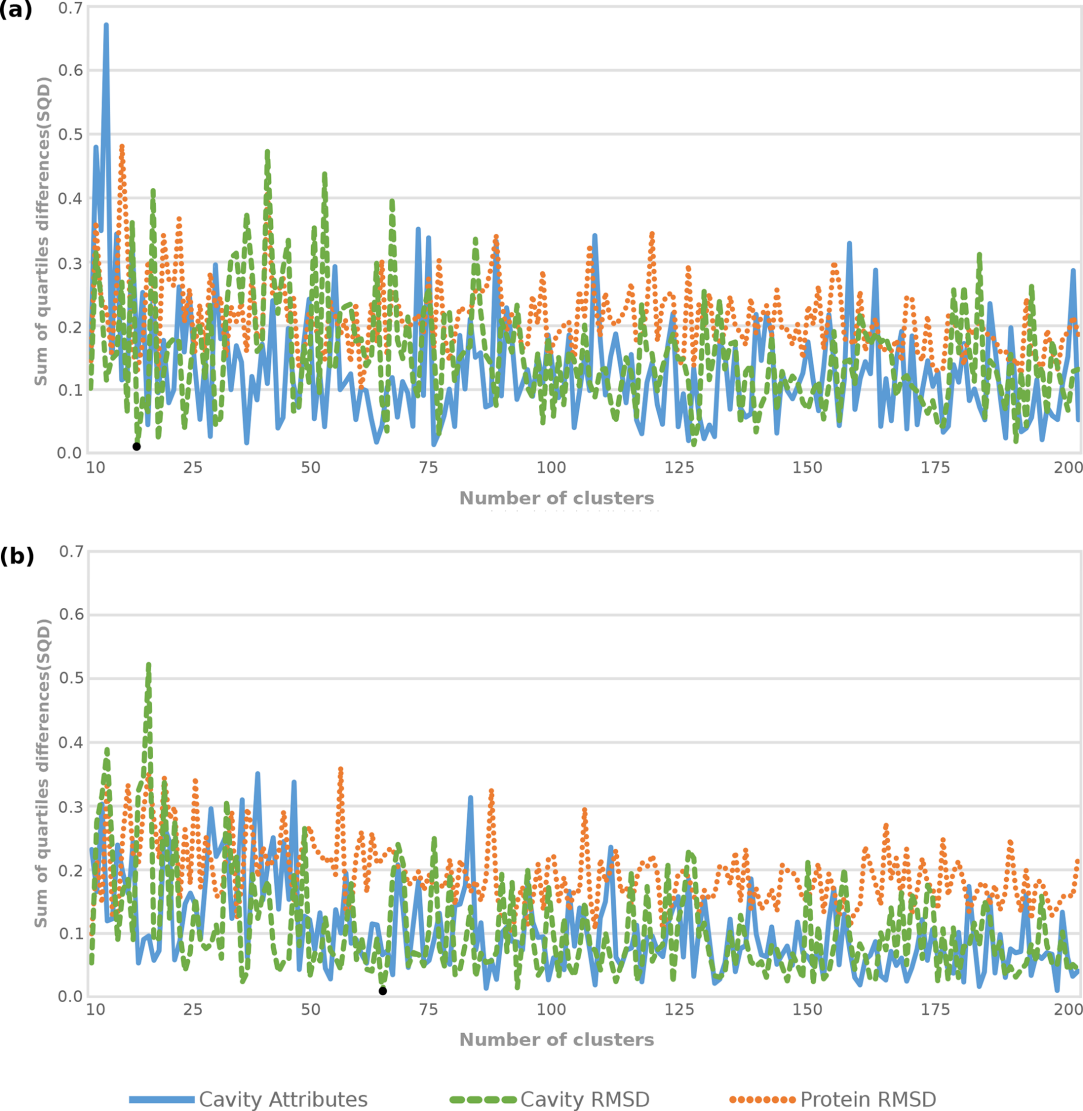
Comparative performance of partitioning clustering methods for the three data sets under study. Variations in the SQD values as a function of the number of clusters for *k*-means and *k*-medoids are showed in the graphs (a) and (b), respectively. The black points identify the optimal partitioning solutions.

**Fig 5:**
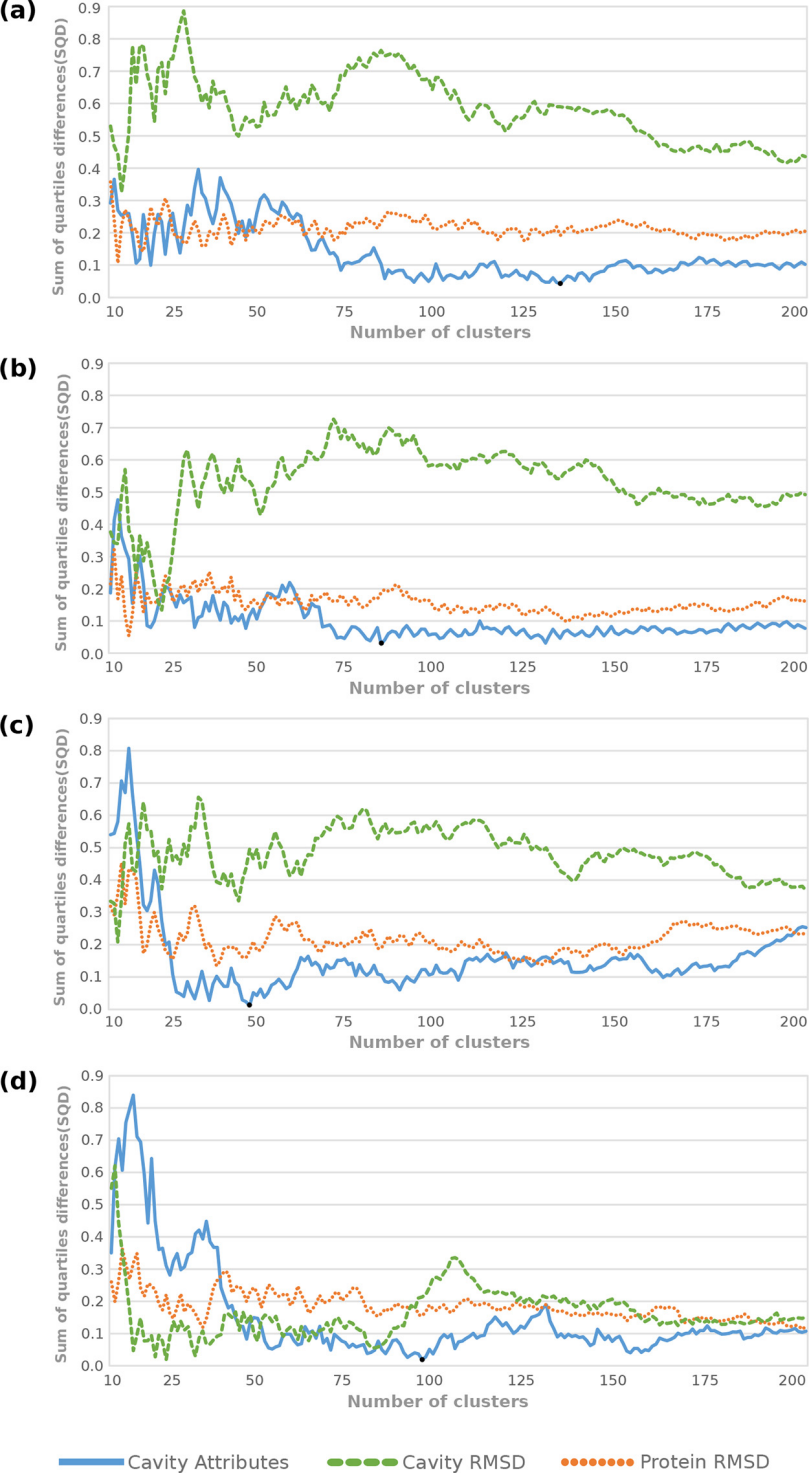
Comparative performance of hierarchical agglomerative clustering methods for each of the three data sets generated from the conformations of the MD trajectory. The SQD values as a function of the number of clusters for, UPGMA, WPGMA, Complete and Ward’s methods are showed in the graphs (a), (b), (c) and (d), respectively. The black points identify optimal partitioning solutions.

To support the second reason above described, we analyzed and compared all clustering solutions taking into account the level of coverage reached by them in terms of dispersion and MD trajectory representativeness. The dispersions among the partitions generated from 10 to 200 clusters were analyzed by assessing the SQD values (Eq 10). The resulting SQD values by clustering method for Attribute, Cavity RMSD and Protein RMSD data sets are in S1, S2 and S3 Tables, respectively. While Figs 4 and 5 show the SQD values as a function of the cluster count, Table 3 shows statistical assessments from the optimal partitions (lowest SQD values) for every clustering method.

**Table 3:** Statistical evaluations for the optimal partitioning solutions obtained from the best partitions (lowest SQD value) of every clustering method. Third column indicates the number of medoids used in the statistical assessments. Average, standard deviation and variance were calculated for each set of medoids based on predicted FEB values. Last row indicates the statistical values for the MD’s full trajectory.

Clustering Method	Data Set	*k* cluster	SQD	Average	Standard Deviation	Variance
k-means	Protein RMSD	19	0.01	-6.61	-0.70	-0.54
k-medoid	Protein RMSD	66	0.01	-6.63	-0.70	-0.55
UPGMA	Cavity Attribute	133	0.04	-6.58	-0.72	-0.56
WPGMA	Cavity Attribute	84	0.03	-6.59	-0.73	-0.58
Complete	Cavity Attribute	48	0.01	-6.59	-0.69	-0.51
Ward’s	Cavity Attribute	95	0.01	-6.60	-0.68	-0.51
-	MD trajectory	20,000	0.00	-6.58	-0.72	-0.57

Fig 4 shows the unbalanced SQD values generated by the partition-based clustering algorithms. Even though k-means and *k*-medoids methods are able to reach low SQD values, they present the statistical values, which were also calculated based on FEB values, far from those found in the MD’s full trajectory (Table 3). For instance, Fig 4a and 4b show Cavity Attributes and Protein RMSD data sets having better SQD values than Cavity RMSD data set for both partitioning methods. However, Table 3 indicates the high difference on the average and variance between the best partitions from these methods and the MD’s full trajectory, which in turn reduces favorable representativeness to the MD trajectory. These inaccurate values can be explained by the fact that partitioning methods work well for finding spherical-shaped clusters in small to medium-sized dataset [43]. The practical consequence of this is represented by the graphs from Fig 4, where the lines come up and down and show slight differences in the SQD values between the data sets as the number of clusters varies.

Unlike partition-based clustering methods, hierarchical algorithms appear to outperform the partitioning for all data sets and present more stable SQD values after a certain number of clusters (Fig 5). As stated earlier, small numbers of medoids are unable to provide large width and are therefore not able to achieve good level of similarity to the quartile values of the MD’s full trajectory. This is evidenced by the high SQD values in the beginning of the graphs (a), (b) and (c) from Fig 5. The Ward’s method presents low SQD values in partitions with 16, 22 and 25 clusters for the Protein RMSD data set. The average and variance values of medoids from these partitions are: -6.62 and -0.51 for k = 16; -6.62 and -0.52 for k = 22; and -6.61 and -0.53 for k = 25. Similar to partitioning methods, these statistical values are far away from the same statistical values found in the MD trajectory and we may conclude that such solutions are unable to become an ensemble of representative MD conformations.

Comparing the performance from hierarchical clustering methods, Fig 5 shows that Ward’s method improved the SQD values for all data. This method also outperforms the affinity with the MD’s full trajectory for the Protein RMSD data set, which in turn shows the worst solutions on other hierarchical methods. Even though Ward’s method presents the best results for all data sets, Table 3 shows the low variance achieved by its best partitioning, i.e. -0.51 for Ward’s solution against -0.57 for the MD trajectory. Jain and Dubes [45] observe that Ward’s method works better for clustering functional data, particularly that are periodic tendencies in the data, but because distance is measured equally in all directions the clusters tend to be spherical—the sum of squares criterion tends to merge small clusters given the same amount of separation. As k-means algorithm, Ward’s method also is able to reach similarity dispersion, but incapable of achieving the similar central tendency undertakes for the entire ensemble of MD conformations.

As expected, the partitions from Cavity Attributes data set appear to accurately determine crucial changes that occur in the substrate-binding cavity of the MD conformations under study. Fig 5 evidences this statement by drawing the Cavity Attributes analyses (blue lines) with lower SQD values and Table 3 indicates the statistical significance for the hierarchical methods regarding to the FEB values. Even though Fig 5 indicates that UPGMA, WPGMA and Complete algorithms are well suited for clustering Cavity Attributes and Cavity RMSD data sets, it does not mean that both data sets have the best representativeness for the MD trajectory since the smallest SQD values are reached by the Cavity Attributes data set only. It means that the black points, which denotes the best solutions, are in the blue line and far from the Cavity RMSD data set line.

### The representative ensemble of MD receptor conformations

One of the major challenges to perform virtual screening experiments on FFR models is to assess to what extent the MD sampling can cover all or most of the binding cavity conformations realized in nature [9, 52]. According to Zhang et al. [33], we should take as many structures as possible to find the most representative conformations. By contrast, Landon et al. [13] showed that a small number of representative conformations could support the development of high-affinity inhibitors capable of binding hot spot regions. Although previous studies apply different techniques to determine an optimal number of representative MD conformations, we validate our solution by examining the level of coverage from the entire MD ensemble based on the results obtained from the cross docking experiments—as we intend to distill crucial changes that occurs within the substrate-binding cavity of the MD trajectories. For that, we statistically evaluated the optimal partitioning solution by accessing the binding cavity of representative MD conformations (medoids) generated by six different clustering methods.

As previously stated, hierarchical clustering methods outperform the clustering solutions for the Cavity Attributes data set, showing statistical values similar to the 20 ns InhA MD trajectory and lowest SQD values. Latter reason is determinant to decide which solution represents the original MD trajectory more accurately. Table 3 shows that Complete and Ward’s methods have the best SQD value for Cavity Attributes data set (SQD = 0.01). Complete method has the average and standard deviation slightly nearest to the MD’s full trajectory and its best solution encloses less medoids than Ward’s method. Bearing this in mind, we defined that the partitioning with 48 clusters from Complete method, that corresponds to 0.01 SQD value, is the optimal solution for representing the MD trajectory under study.

**Fig 6:**
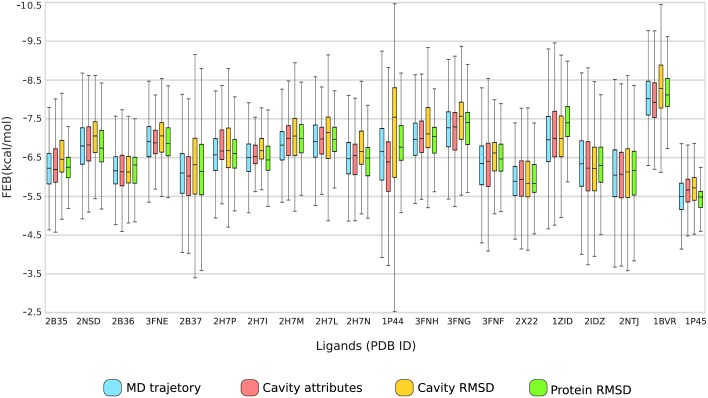
The ensemble of representative MD conformations generated from the best partitioning solution. The boxplot represents the trends in docking result changes in median FEB per data set and per ligand. The data sets are represented by different colors, where, pink, yellow, and green are the data sets used in the clustering experiments and blue is the original simulation.

We generated the boxplot graph to compare the data distribution between the optimal partition solution and the MD’s full trajectory based on predicted FEB values from cross-docking experiments. Fig 6 represents the conformations range from the first quartile to the third quartile with the median FEB values denoted by the black line across the central box region. The bottom and top whiskers each extend an additional 1.5 times the distance from the median to the first and third quartiles, but they are truncated to the minimum and maximum data values, respectively [53]. The coverage comparison between the representative ensembles produced by Cavity Attributes, Cavity RMSD and Protein RMSD data set and the MD’s full trajectory for the 20 ligands analyzed is depicted in Fig 6.

As can be seen from Fig 6, the boxplots from Cavity Attributes report dispersions and central tendencies noticeably more similar to the entire MD ensemble than the other two data sets (Cavity RMSD and Protein RMSD). It can therefore be assumed that the representative ensemble generated by the proposed data set reproduces a fairly good number of docking poses obtained when the ligands tested were docked into the MD’s full trajectory. Remember that each ensemble of representative MD trajectory contains 48 conformations. However, only the Cavity Attributes data set successfully embodied the highest percentage of different changes in the binding cavity for all ligands tested. At the same time, it must be pointed out that this small set of ligands is used to validate our approach for clustering MD trajectories relied on features from the substrate-binding cavity. The level of convergence achieved by using this new representative ensemble may change depending on the ligands. For instance, Fig 6 shows the poor convergence reached by the ligands from 3FNE, 2H7P, 2H7I, 2H7L and 1P44 PDB ID on the MD trajectory. According to our findings, the reduced ensemble of MD conformations was capable of representing all possible moves within the substrate-binding cavity for 75% of tested ligands. It means that, our approach performed successfully as the ensemble of representative MD conformations represents a high level of docking results. Further, we identified that three of the remaining 25% ligands contain the median closely to the MD trajectory (PDB IDs: 3FNE, 2H7I and 2H7L), one represents the best FEB values (PDB ID: 2H7P) and only one covers the worst FEB values (PDB ID: 1P44). Therefore, we concluded that properties from the substrate-binding cavity and the functionality offered by Complete hierarchical algorithm can overcome the problem of reducing the FFR model dimension to a manageable size, keeping the most meaningful information to find novel inhibitors during a docking search. The reduced ensemble is called a RFFR model [10, 48].

## Discussion

We have presented a strategy to generate ensembles of representative MD conformations that are more sensitive to changes in the substrate-binding cavity properties than the widely used RMSD approaches. This strategy uses two partitioning clustering methods (k-means and k-medoids) and four agglomerative hierarchical clustering methods (complete linkage, UPGMA, WPGMA and Ward’s). We use them in order to compare and analyze the quality of partitioning outcomes between the binding cavity data set that we are proposing and two different data sets composed by pairwise RMSD distances. To provide the optimal ensemble of representative MD conformations, we obtained the FEB values from docking experiments with 20 known inhibitors of the InhA enzyme by which we identified optimal partitions by statistical assessments and calculated their percentage of similarity with the original MD trajectory.

The results for hierarchical algorithms highlighted their main advantages, i.e. they are more versatile and embed flexibility regarding seeking a proper level of granularity. Comparing the performance from clustering methods, Fig 5 shows that UPGMA, WPGMA and Complete are good methods for clustering kinds of data sets similar to Cavity Attributes and Cavity RMSD while Ward’s methods can be considered a good solution for all data set. However, the ability of Ward’s in grouping objects that are as homogeneous as possible ended in partitions with central tendencies considerably far from that found in the MD’s full trajectory. Further, the high cohesion in the clusters generated from UPGMA and WPGMA methods were unable to reach low SQD values and number of clusters. Complete method looks for maximum distance to merge a new object in a cluster and therefore it becomes more susceptible to noise and outliers. Remember that the first 500 conformations from the MD trajectory were eliminated as they constitute the equilibration phase. For this reason Complete method shows the lowest SQD values and number of clusters for the Cavity Attributes. Hence, we conclude that due to the farthest neighbor method the representative ensemble of MD conformations is composed by medoids belonging to compact clusters of approximately equal diameters.

The complexity of clustering algorithms is strongly related to the number *n* of data objects and the number *k* of clusters [54, 55]. From all experiments, CLARA was the algorithm that required the longest execution time, considering an experiment when the number of partitions starts from 2 until 200. The time noticeably increased since the size of the sample grows proportionally to the number of clusters. It happens on the account of *k*-medoids is more robust in the presence of noise and outliers. The complexity to compute and select a new medoid from representative objects by PAM algorithm is O(k(n-k)^2^). Algorithms from hierarchical agglomerative methods are in second position. They are expensive in terms of their computational and storage requirements [42]. Agglomerative methods compute the proximity matrix that needs O(n^2^) time to store and keep track of the clusters. The total time required for these algorithms is O(n^2^ ×log_g_ n) where log_n_ is the additional complexity of keeping data in a sorted list. In contrast to the hierarchical algorithms that have the quadratic asymptotic running time with respect to the number of objects, *k*-means produces a number of partitions for every *k* in a linear time complexity with respect to any aspect of the problem size [54]. The complexity of *k*-means algorithm is O(nkh), where the number of clusters (k) and the number of interactions (h) are usually less than the number of objects (n).

Several works explore the relative accuracy of various clustering algorithms in extracting the right number of clusters from generated data [43]. According to Hartigan et al. [18], we cannot point the best clustering method since different approaches are right for different purposes. Chen and Lonardi [15] say that the more popular methods for clustering MD conformations are agglomerative hierarchical clustering since its linkage method is able to use the attributes for describing the chemical structures. More specifically, linkage is the only method able to calculate the dissimilarities between two clusters of chemical structures using Euclidean distance. Alternatively, Shao et al. [16] found that UPGMA, *k*-means, and SOM outperformed COBWEB, Bayesian, and other hierarchical clustering methods by using the pairwise RMSD distance as measure of similarity. Although our analyses also show hierarchical agglomerative methods as the best choices for all data sets, the *k*-means and *k*-medoids algorithms appear as the worst choice for all data sets. Each study has its own way to generate data and to identify the best clustering algorithm and, therefore, comes with its own advantages and drawbacks. Indeed, an appropriate solution depends on a given analysis or application scenario, so data collection, data representation, and interpreting the clusters found are crucial for selecting a clustering strategy [45, 55].

## Conclusions

The work we present here analyzes and combines clustering partitions using three different data sets in order to reduce the structural redundancy in a 20 ns MD trajectory of a target protein receptor. Previous studies tackled this computational issue using only the RMSD measure of similarity [13, 16, 17]. The present study, in addition to investigating RMSD-based clustering, also provides a novel measure of similarity, which is based on features from the substrate-binding cavity (pairwise RMSD, volume and number of heavy atoms). It addresses the high computational cost involved in using MD ensembles for performing virtual screening of large libraries. We learned that the use of binding cavity properties for clustering MD trajectory is an efficient method to distill significant conformational flexibility within the receptor binding cavity. The chosen properties also outperformed other RMSD measures of similarity. This methodology can be extended to other proteins/receptor, as long as the binding pocket from the FFR model is known in advance. Further applications may include the investigation of ensembles of MD conformations from other target receptor enzymes, as well as with longer MD simulation trajectories. Future directions involve the extension of this approach to the exploration of virtual libraries of compounds where the ensemble of representative MD conformations, shaped by properties of the substrate-binding cavity, can be investigated more effectively.

## Supporting Information

S1 Dataset Protein RMSD data set. (XLS)

S2 Dataset Cavity RMSD data set. (XLS)

S3 Dataset Cavity Attributes data set. (XLS)

S1 Fig Analysis of substrate-binding cavity volumes along 20 ns MD simulation trajectory obtained from CASTp. In red the substrate-binding cavity average volume at 1,236.9 Å^3^. The higher (yellow line) and lower (green line) volumes of this binding cavity achieved from crystal structures were taken at 2,032.8 Å^3^ (PDB ID: 4OXN) and 445.1 Å^3^ (PDB ID: 2B37), respectively.
(PNG)

S1 Table SQD values from Cavity Attributes data set by partitioning and clustering method used. (DOCX)

S2 Table SQD values from Cavity RMSD data set by partitioning and clustering method used. (DOCX)

S3 Table SQD values from Protein RMSD data set by partitioning and clustering method used. (DOCX)
