# Supplementary figures and images for: An Effective Approach for Clustering InhA Molecular Dynamics Trajectory Using Substrate-Binding Cavity Features
Source: PLoS One. 2015 Jul 28;10(7):e0133172. doi: 10.1371/journal.pone.0133172 (PMC4517875; doi:10.1371/journal.pone.0133172)

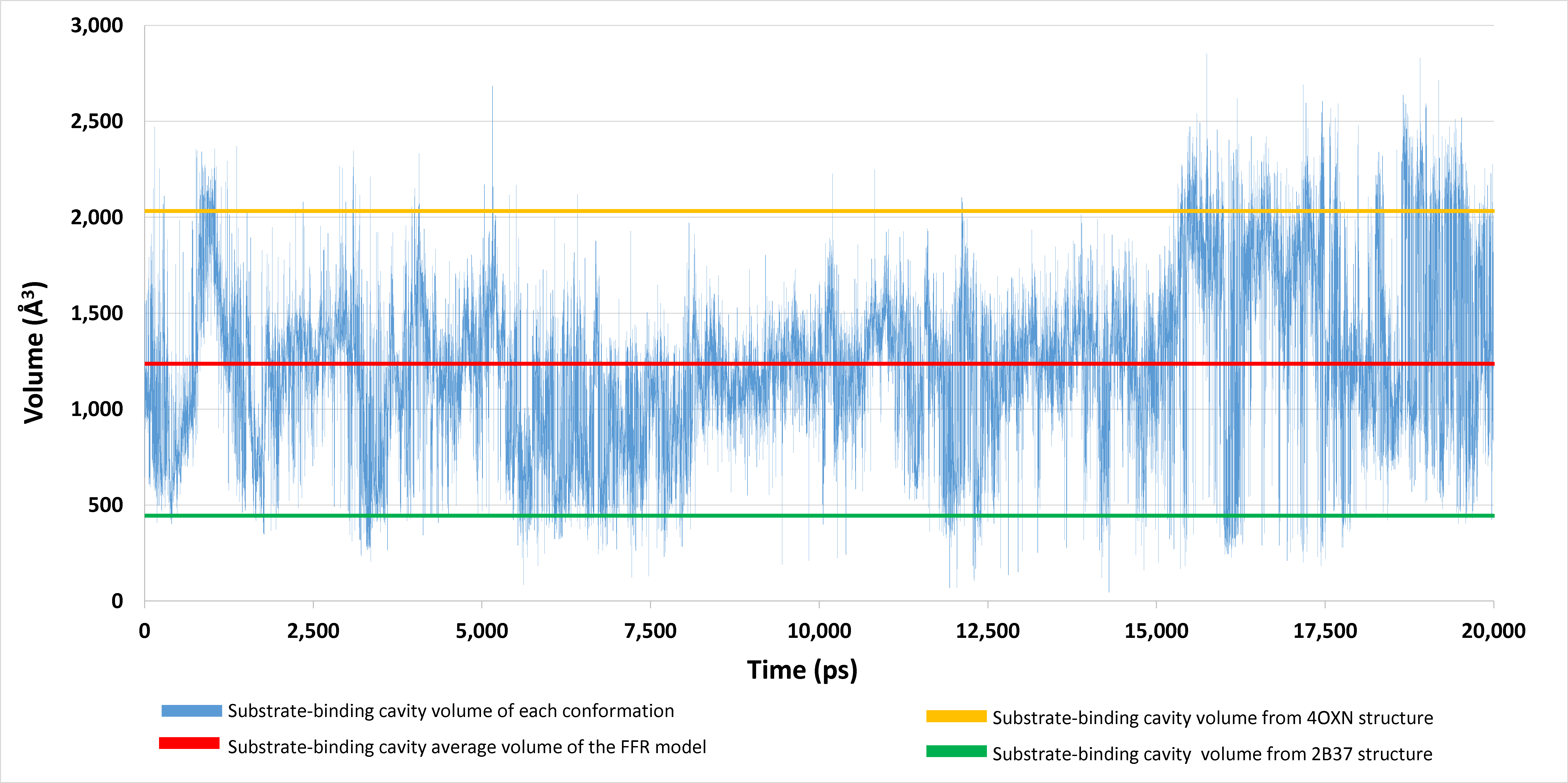

Supplement: S1 Fig — In red the substrate-binding cavity average volume at 1,236.9 Å3. The higher (yellow line) and lower (green line) volumes of this binding cavity achieved from crystal structures were taken at 2,032.8 Å3 (PDB ID: 4OXN) and 445.1 Å3 (PDB ID: 2B37), respectively. (PNG) [file pone.0133172.s004.png]
